# Ten-Year Cardiovascular Disease Risk Among Employees Aged 40 Years and Older at the University of Sharjah: A Cross-Sectional Study

**DOI:** 10.7759/cureus.105045

**Published:** 2026-03-11

**Authors:** Mohamed M Alhariri, Rama N Alshanawani, Rana T Hamada, Fatma F Lootah, Omar J Faraj, Rizwan Qaisar

**Affiliations:** 1 Cardiology, College of Medicine, University of Sharjah, Sharjah, ARE; 2 Basic Medical Sciences, College of Medicine, University of Sharjah, Sharjah, ARE

**Keywords:** cardiovascular diseases, lifestyle behaviors, occupational health, physical activity, risk factors, smoking

## Abstract

Background: Atherosclerotic cardiovascular diseases (ASCVDs) are a leading cause of mortality globally and in the United Arab Emirates. Despite regional studies on the ASCVD risk, no prior study has assessed the ASCVD risk within the academic community of the University of Sharjah. This study aimed to estimate the 10-year predicted ASCVD risk among university employees aged ≥40 years and to identify associated lifestyle risk factors.

Methods: In this cross-sectional study, a stratified random sample of 124 employees aged 40 or older without a prior history of ASCVD was enrolled. The primary outcome was the 10-year ASCVD risk score calculated using the Mayo Clinic ASCVD risk calculator (Mayo Clinic, Rochester, MN). Data were analyzed using IBM Statistical Package for the Social Sciences Statistics version 22.0 (IBM Corp., Armonk, NY). Associations between ASCVD risk scores and lifestyle factors were assessed using Mann-Whitney U and Kruskal-Wallis tests, with statistical significance set at p < 0.05.

Results: Among the 124 participants included in the study (59.7% male and 40.3% female participants), the mean 10-year predicted ASCVD risk was classified as high at 37.16% (standard deviation ± 17.47). ASCVD risk scores were significantly higher among smokers compared with nonsmokers (48.6% vs. 32.7%, p < 0.001) and similarly elevated among participants with diabetes compared with nondiabetics (55.1% vs. 33.9%, p < 0.001). Physical activity level was also significantly associated with ASCVD risk (p = 0.009), while fruit and vegetable intake, saturated fat intake, daily sitting duration, and working hours showed no significant association (p > 0.05).

Conclusions: Employees aged ≥40 years at the University of Sharjah demonstrate a markedly high predicted 10-year ASCVD risk. Smoking, diabetes, and physical inactivity were identified as key modifiable risk factors associated with higher predicted ASCVD risk. These findings underscore an urgent need for targeted workplace health promotion interventions to reduce cardiovascular risk in this population.

## Introduction

Atherosclerotic cardiovascular diseases (ASCVDs) remain the leading cause of mortality worldwide, accounting for an estimated 17.9 million deaths annually [1]. In the United Arab Emirates (UAE), the burden is substantial, with high age-standardized mortality rates reported nationally [2]. This may potentially be related to lifestyle changes, including reduced physical activity and dietary shifts, associated with rapid socioeconomic development [2,3]. Importantly, up to 70% of ASCVD cases are preventable through modification of behavioral risk factors [4], highlighting the need for early risk identification and targeted prevention.

The UAE reports higher ASCVD mortality rates compared to many Western countries [2]. In comparison, mortality rates are lower in North America and Western Europe [1]. These disparities highlight the importance of context-specific research to better understand cardiovascular risk within local populations. ASCVD risk factors include behavioral and clinical conditions such as physical inactivity, unhealthy diet, smoking, diabetes, age, and family history that significantly increase the likelihood of cardiovascular events [4]. Numerous studies have demonstrated that adherence to healthy lifestyle practices, particularly regular physical activity, has been shown to reduce ASCVD incidence in a dose-dependent manner [5-7].

Academic institutions represent a distinct occupational environment where employees may be exposed to lifestyle-related cardiovascular risks: prolonged sedentary work, high occupational stress, and limited opportunities for physical activity, all of which can adversely affect cardiovascular health [8]. Regional data support this concern; a study conducted in Saudi Arabia found that 30% of university faculty members had one or more cardiovascular risk factors [9], suggesting that academic staff may constitute an underrecognized high-risk group.

Despite this, the cardiovascular risk profile of employees at the University of Sharjah remains uncharacterized. Therefore, the primary objective of this study was to estimate the 10-year predicted ASCVD risk among employees aged ≥40 years using the pooled cohort equations through the Mayo Clinic ASCVD Risk Calculator (Mayo Clinic, Rochester, MN) [10,11]. The secondary objective was to examine the association between predicted ASCVD risk and key modifiable lifestyle factors, including smoking, physical activity, working hours, and dietary habits, as well as to determine the prevalence of these behaviors among employees. We hypothesized that diabetes, smoking, physical inactivity, prolonged sitting, and long working hours would be associated with higher calculated 10-year ASCVD risk among employees aged ≥40 years.

## Materials and methods

Study design and setting

This cross-sectional study was conducted at the University of Sharjah, an urban academic institution where most employees work in predominantly sedentary office-based roles with limited on-campus opportunities for structured physical activity or access to nutritious foods. These environmental and geographic factors provide context for the lifestyle behaviors assessed, including physical inactivity, dietary habits, and working hours. The study design allowed estimation of the prevalence of these habits and their associations with ASCVD risk without long-term follow-up, providing efficient insights for occupational health interventions.

Ethical considerations

Ethical approval was obtained from the University of Sharjah Research Ethics Committee (approval no. REC-23-02-19-07-S). Written informed consent was obtained from all participants prior to enrollment in the study, and all procedures adhered to the principles outlined in the Declaration of Helsinki.

Sampling and sample size

A total of 124 employees participated in the study. Inclusion criteria included employees of the University of Sharjah aged 40 years or older, in line with the American College of Cardiology/American Heart Association (ACC/AHA) guideline recommending initiation of a 10-year ASCVD risk assessment at age 40 [4]. Exclusion criteria included a preexisting history of ASCVD (e.g., myocardial infarction, stroke, transient ischemic attack, and peripheral artery disease) or prior cardiovascular procedures (e.g., angioplasty, stent placement, and abdominal aortic aneurysm repair).

To strengthen the methodological rigor and precision of the study, the minimum sample size was determined prior to using a standard approach for estimating a population proportion with 95% confidence. Since no prior studies have reported the prevalence of ASCVD in our target population, we assumed an expected prevalence of 50%, which ensures a sufficiently large sample size and reduces the chance of underestimation. A margin of error of 10% was chosen to balance precision with practical feasibility. This process resulted in a minimum requirement of 100 participants.

Stratified random sampling was employed to ensure proportional representation of academic staff, administrative staff, and security personnel. Participants were first grouped by occupation (strata), after which a complete list of eligible individuals within each stratum was obtained from institutional rosters. Participants were then randomly selected within each stratum using a computer-generated random number sequence to minimize selection bias. Eligible employees were subsequently invited to participate via institutional email. The invitation outlined the study objectives and procedures, and stated that participation involved completing a questionnaire and undergoing blood pressure (BP) measurement. Participation was voluntary.

Data collection methods

Data were collected using a structured self-administered electronic questionnaire, which comprised three sections: 1) demographics, 2) medical and family history, and 3) lifestyle behaviors, and was developed based on the input variables required for the Mayo Clinic ASCVD Risk Estimator [11], including age, sex, race, total cholesterol, high-density lipoprotein (HDL) cholesterol, systolic BP, hypertensive treatment, diabetes status, and smoking status, as well as components of the World Health Organization Stepwise approach to noncommunicable disease risk factor surveillance [12].

Content validity was assessed by two public health specialists to ensure clarity, relevance, and appropriateness of all items. Prior to distribution, the questionnaire was reviewed by the research team for consistency, structure, and comprehensibility. The estimated completion time was seven to ten minutes.

Written informed consent was obtained electronically before participants completed the questionnaire. Although the questionnaire was administered electronically, it was completed in person in the presence of a member of the research team to address any queries. All responses were submitted electronically and stored securely for subsequent analysis.

Demographic information, smoking status, physical activity levels, and medical history were self-reported. Lipid profile parameters (total cholesterol and HDL cholesterol) were also self-reported by participants based on their most recent laboratory test results. In contrast, BP was measured directly by the research team using a validated digital sphygmomanometer in accordance with World Health Organization guidelines [12]. All measurements were performed by trained members of the research team using the same validated device to ensure consistency and standardization. Participants were seated and rested for at least five minutes prior to measurement. Two readings were taken at one-to-two-minute intervals, and the average of the two readings was recorded for analysis. Given the reliance on self-reported clinical data, such as lipid values, the possibility of recall bias or misclassification cannot be excluded, which may have influenced the precision of the calculated ASCVD risk estimates.

The 10-year predicted ASCVD risk was calculated using the pooled cohort equations developed by the ACC/AHA as described by Goff et al. [10]. Risk estimation was implemented using the Mayo Clinic ASCVD Risk Estimator tool (Mayo Clinic, Rochester, MN) [11], which applies the pooled cohort equations algorithm.

Statistical analysis

Data were coded, cleaned, and analyzed using IBM Statistical Package for the Social Sciences Statistics for Windows, version 22.0 (IBM Corp., Armonk, NY). Descriptive statistics included mean ± standard deviation (SD) for continuous variables and frequencies (%) for categorical variables.

Normality of the ASCVD risk score was assessed using the Shapiro-Wilk test. Because the risk scores were not normally distributed, nonparametric tests were used for bivariate analyses: the Mann-Whitney U test for binary variables and the Kruskal-Wallis test for variables with more than two categories.

A simple linear regression was performed to assess the association between age (continuous) and ASCVD risk (continuous). Statistical significance was set at p < 0.05 for all analyses. Due to the modest sample size, multivariable regression analyses adjusting for potential confounders were not performed and should be considered in future studies. Similarly, effect sizes were not calculated, as estimates could be unreliable; this limitation is acknowledged to ensure transparency regarding the precision of the estimated associations.

## Results

The mean 10-year predicted ASCVD risk for the cohort was 37.2% (SD ± 17.47), placing the average participant in the high-risk category. Furthermore, 84% (n = 104) of participants were classified as having a high-to-very-high predicted risk (≥20%). The cohort (n = 124) had a mean age of 49.8 years (SD ± 6.7), was predominantly male (59.7%), Arab (71.0%), and consisted mainly of academic staff (64.5%). Full demographic and clinical characteristics, along with the prevalence of lifestyle behaviors assessed in this study, are presented in Table 1.

**Table 1:** Baseline characteristics and demographics of the study population ASCVD: atherosclerotic cardiovascular disease; SD: standard deviation

Characteristic	Values
Age (years), mean ± SD (range)	49.75 ± 6.71 (40-66)
<47, n (%)	44 (35.5%)
47-52, n (%)	43 (34.7%)
>52, n (%)	37 (29.8%)
Gender, n (%)	
Male	74 (59.7%)
Female	50 (40.3%)
Ethnicity, n (%)	
Arab	88 (71.0%)
Non-Arab	36 (29.0%)
Marital status, n (%)	
Married	118 (95.0%)
Single	6 (5.0%)
Education level, n (%)	
PhD or higher	26 (21.0%)
Master’s degree	23 (18.5%)
Bachelor’s degree	75 (60.5%)
Working position, n (%)	
Academic staff	80 (64.5%)
Security staff	26 (21.0%)
Office staff	18 (14.5%)
Mean calculated ASCVD risk, mean ± SD	37.16 ± 17.47
Family history of ASCVD, n (%)	
Yes	82 (66.1%)
No	42 (33.9%)
Blood pressure, n (%)	
Normal	67 (54.0%)
Slightly above normal	42 (33.9%)
High	8 (6.5%)
Antihypertensive medication, n (%)	
Yes	33 (26.8%)
No	91 (73.2%)
Smoking (≥100 lifetime cigarettes), n (%)	
Yes	35 (28.2%)
No	89 (71.8%)
Physical activity, n (%)	
Not physically active	33 (26.6%)
Moderately active	44 (35.5%)
Vigorous activity	47 (37.9%)
Fruits and vegetables intake, n (%)	
≥5 servings/day	20 (16.1%)
2-4 servings/day	74 (59.7%)
0-1 serving/day	30 (24.2%)
Saturated animal fat intake, n (%)	
≥2 servings/week	63 (50.8%)
0-1 serving/week	61 (49.2%)

**Figure 1:**
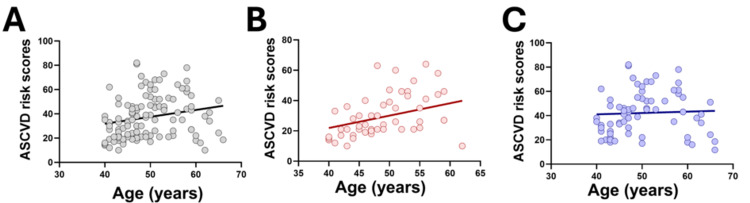
Relationship between age and calculated ASCVD risk score in the overall cohort and stratified by sex (A) Association between age and ASCVD risk score in the overall cohort. (B) Association between age and ASCVD risk score in women. (C) Association between age and ASCVD risk score in men ASCVD: atherosclerotic cardiovascular disease

A significant positive association was observed between increasing age and calculated ASCVD risk (r² = 0.047, p < 0.05) (Figure 1A). When stratified by sex, this association remained statistically significant only among women (r² = 0.126, p < 0.05) (Figure 1B), whereas in men the relationship did not reach statistical significance despite a positive trend (r² = 0.07, p = 0.21) (Figure 1C).

**Figure 2:**
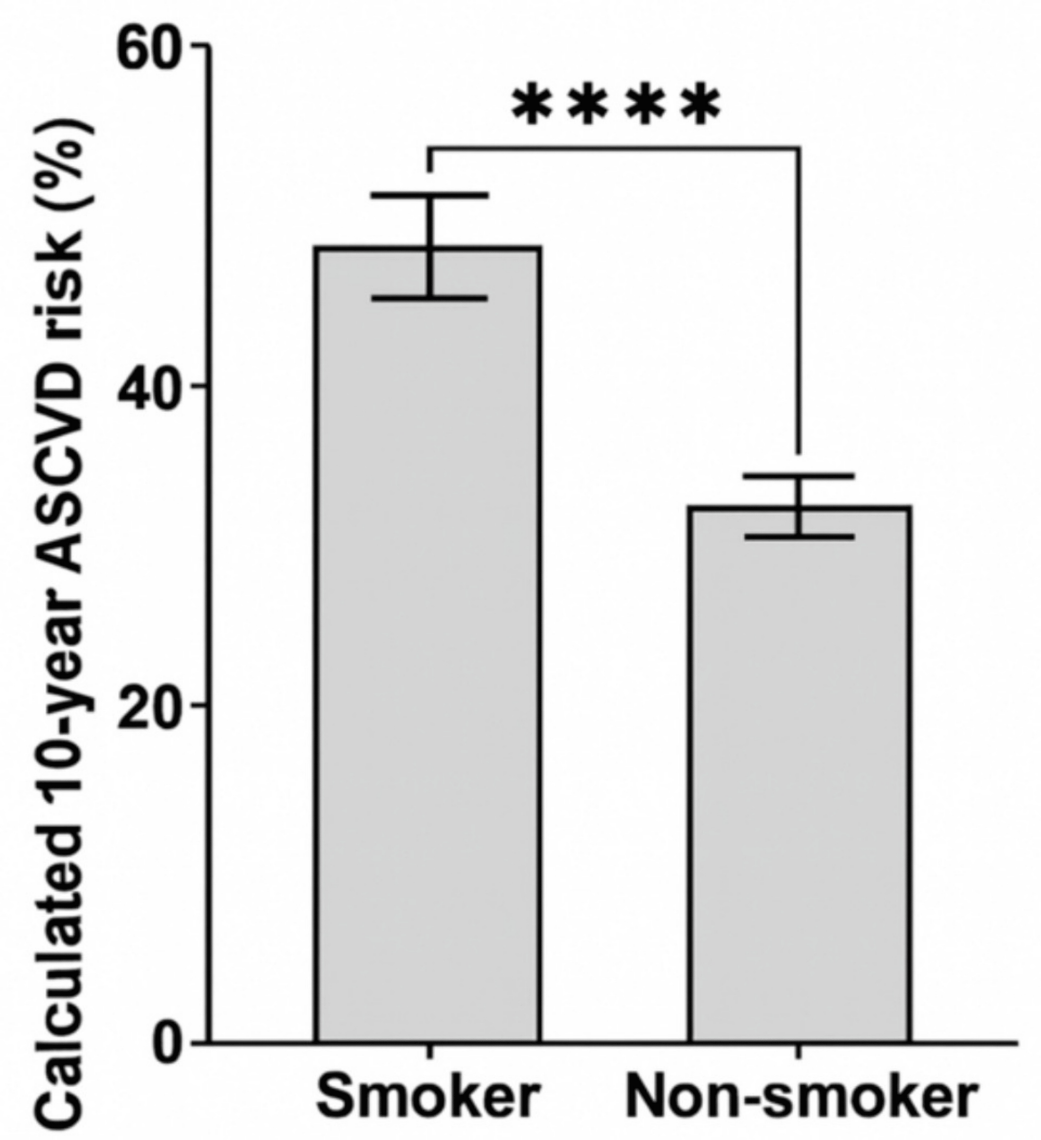
Distribution of calculated 10-year ASCVD risk according to smoking status ASCVD: atherosclerotic cardiovascular disease ^****^Statistical significance at p < 0.001

Smoking status was significantly associated with ASCVD risk, with smokers exhibiting higher risk scores than nonsmokers (Mann-Whitney U = 776.5, Z = -4.34, p < 0.001) (Figure 2, Table 2). Participants with diabetes also showed significantly elevated risk scores compared with nondiabetic participants (Mann-Whitney U = 360.0, Z = -4.42, p < 0.001). Furthermore, a statistically significant difference in ASCVD risk was observed across physical activity categories (Kruskal-Wallis H(2) = 9.41, p = 0.009) (Table 2).

**Table 2:** Summary of inferential analysis: association between lifestyle factors and calculated ASCVD risk ASCVD: atherosclerotic cardiovascular disease

Lifestyle factor	Comparison grouping	Test used	p value
Smoking status	Yes vs. No (smoked ≥100 cigarettes)	Mann-Whitney U test	<0.001
Diabetes	Yes vs. No (diagnosed with diabetes)	Mann-Whitney U test	<0.001
Physical activity	Not physically active vs. moderately active vs. vigorously active	Kruskal-Wallis test	0.009
Fruits and vegetables	0-1 vs. 2-4 vs. ≥5 servings/day	Kruskal-Wallis test	0.455
Saturated fat	≥2 vs. 0-1 servings/day	Mann-Whitney U test	0.177
Sitting hours	≥5 hours vs. 0-5 hours/day	Mann-Whitney U test	0.818
Working hours	≥8 hours vs. 6-8 hours/day	Mann-Whitney U test	0.254

**Figure 3:**
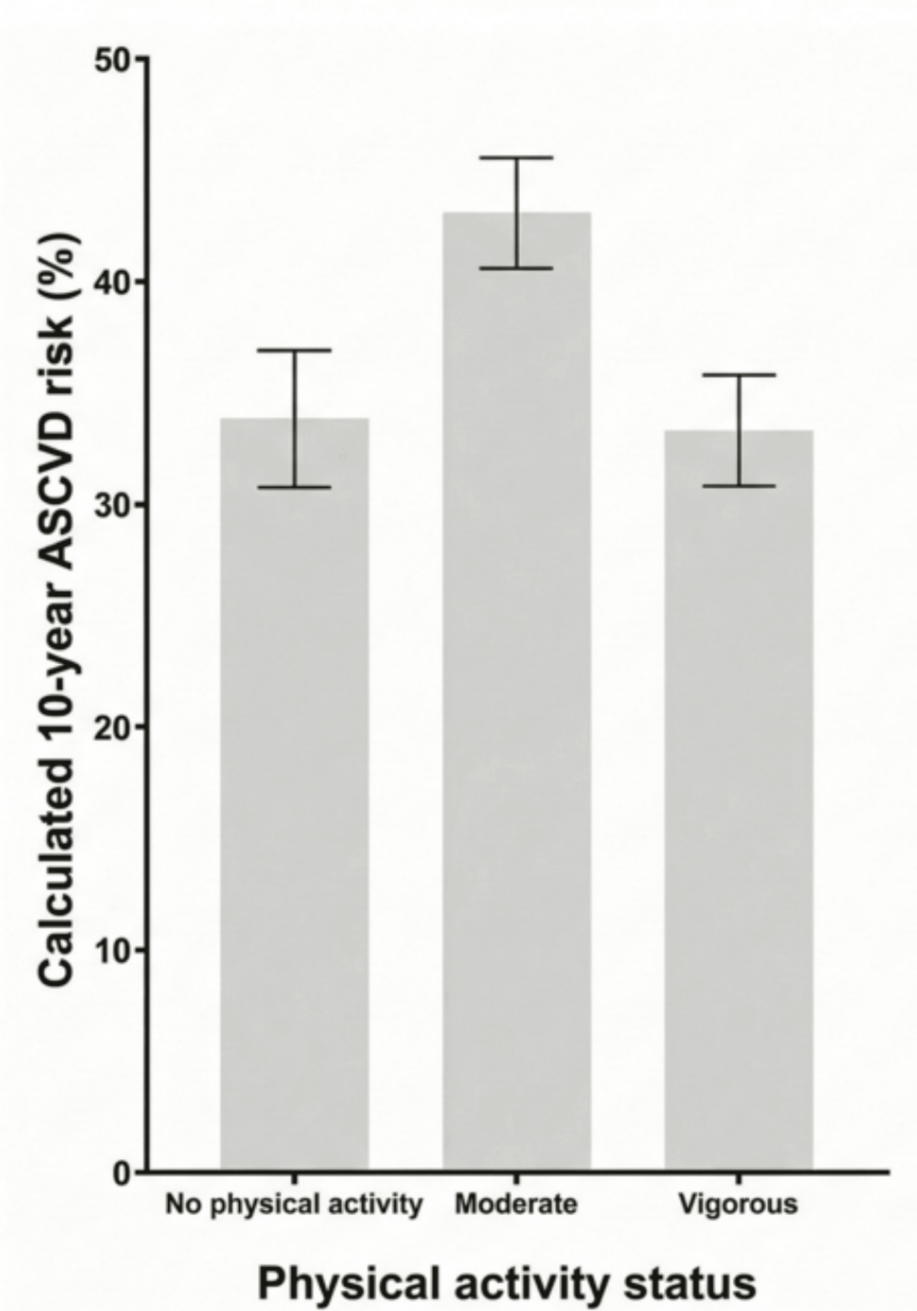
Distribution of calculated 10-year ASCVD according to physical activity status ASCVD: atherosclerotic cardiovascular disease

The distribution of ASCVD risk scores by physical activity level is illustrated in Figure 3. While 30.6% of participants reported engaging in vigorous physical activity and 26.6% reported no physical activity (Table 1), ASCVD risk did not show a linear dose-response pattern across activity groups, with participants reporting moderate physical activity exhibiting higher ASCVD risk scores than both inactive and vigorously active groups.

To further contextualize the magnitude of these associations, mean 10-year ASCVD risk scores were examined across key demographic and lifestyle categories. As shown in Table 3, higher mean risk scores were observed among men, smokers, and participants with diabetes, with additional variation across physical activity categories.

**Table 3:** Mean 10-year ASCVD risk score stratified by key demographic and lifestyle factors ASCVD: atherosclerotic cardiovascular disease; SD: standard deviation

Variable	Category	Mean ASCVD risk	SD	p value
Gender	Male	41.9	17.9	<0.001
	Female	30.1	14.2	
Smoking status	Ever smoker (≥100 cigarettes)	48.6	17.7	<0.001
	Never smoker	32.7	15.3	
Diabetes	Yes	55.0	16.9	<0.001
	No	33.9	15.6	
Fruit and vegetable intake	≥5 servings/day	35.7	14.8	0.533
	2-4 servings/day	38.6	17.9	
	0-1 servings/day	34.6	18.2	
Physical activity	Not physically active	33.8	17.7	0.012
	Moderately active	43.1	17.0	
	Vigorous activity	33.3	16.4	

**Figure 4:**
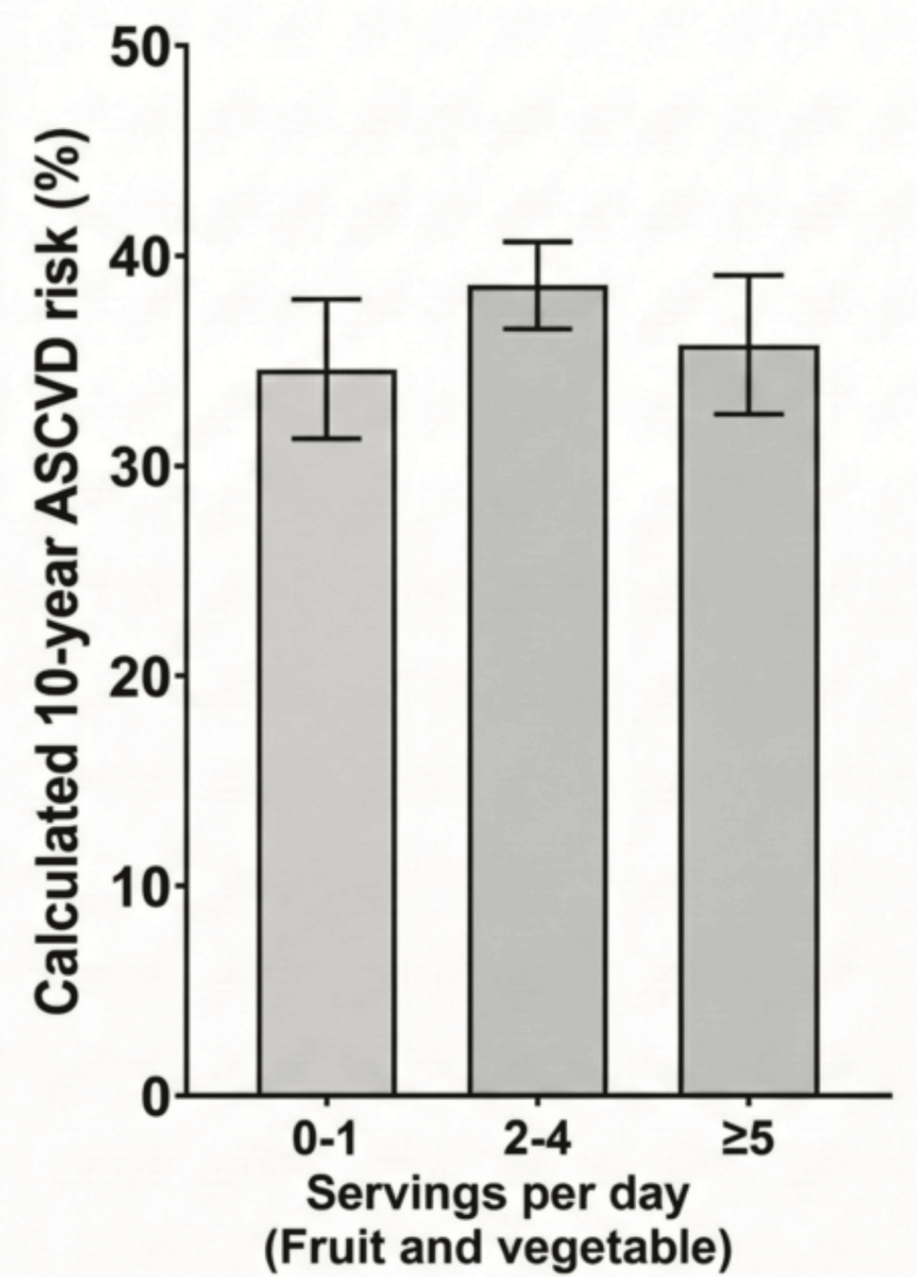
Distribution of calculated 10-year ASCVD risk across categories of fruit and vegetable intake ASCVD: atherosclerotic cardiovascular disease

In contrast, fruit and vegetable intake (H(2) = 1.57, p = 0.455), saturated fat intake (U = 1651.5, p = 0.177), daily sitting duration (H(1) = 0.053, p = 0.818), and working hours (U = 1677.0, p = 0.254) were not significantly associated with the ASCVD risk (see Table 2). The distribution of ASCVD risk across fruit and vegetable intake categories is illustrated in Figure 4, which visually supports the lack of statistically significant differences.

## Discussion

The present study revealed that a substantial proportion of University of Sharjah employees aged ≥40 years exhibited moderate-to-high predicted 10-year ASCVD risk, with a mean risk of 37.2%. This strikingly elevated average risk positions this occupational group well within a high-risk category and highlights an urgent need for targeted preventive strategies. These findings are consistent with the elevated cardiovascular disease burden reported both regionally in the UAE [2] and globally [1]. To our knowledge, this is the first study to quantify predicted cardiovascular disease risk in this UAE academic population, thereby filling an important regional knowledge gap.

The elevated risk profile appears to be associated primarily with age, smoking, and diabetes, which are factors that carry substantial weight within established cardiovascular risk prediction models [4,10]. The strong association between increasing age and predicted risk is expected, given its central role in pooled cohort equations [10]. Although sex-stratified analyses revealed differences in the strength of association between age and predicted risk, prior literature consistently demonstrates sex-related differences in cardiovascular mortality and risk accumulation [13,14]. While men typically demonstrate earlier clustering of cardiometabolic risk factors, women may experience higher risk following menopause, contributing to differences in lifetime cardiovascular risk trajectories [13,14].

Smoking and diabetes, both highly prevalent and modifiable, emerged as key correlates, consistent with established evidence identifying tobacco exposure and diabetes mellitus as major modifiable risk factors for ASCVD [4,6]. The ACC/AHA prevention guidelines emphasize both smoking cessation and glycemic control as cornerstone interventions for reducing cardiovascular risk [4], and large multinational cohort data further confirm the independent contribution of these factors to cardiovascular morbidity and mortality [6].

Although regular physical activity is widely recognized as protective against cardiovascular morbidity and mortality in a dose-dependent manner [5,6], our findings revealed a nonlinear distribution of predicted risk across activity categories. Participants reporting moderate physical activity had a higher predicted risk than those reporting either no activity or vigorous activity. While this pattern does not follow a conventional dose-response gradient, it may reflect residual confounding, misclassification bias, or underlying health status differences among participants who engage in moderate activity due to preexisting health concerns. Prior studies have consistently shown that higher levels of physical activity are associated with reduced cardiovascular mortality and improved metabolic profiles [5,6], suggesting that the observed paradox warrants further investigation rather than contradicting established evidence.

Dietary intake of fruits and vegetables did not demonstrate a statistically significant association with calculated ASCVD risk in this cohort. Although large-scale prospective data support the cardiovascular benefits of higher fruit and vegetable consumption [6], the absence of association in this study may reflect limited dietary variability, self-report bias, or the use of broad intake categories rather than detailed nutritional assessment. Similarly, prolonged sitting duration and working hours were not significantly associated with predicted risk. While meta-analytic evidence suggests that long working hours are associated with increased cardiovascular risk [8], the relatively homogeneous occupational environment of academic staff may have limited variability in exposure, reducing statistical power to detect associations.

The occupational context may further amplify this risk. Academic work is frequently characterized by prolonged sedentary time, psychosocial stress, and time constraints that may limit engagement in preventive health behaviors. While these factors were not independently predictive within our analysis, they likely coexist with established clinical risks to shape long-term cardiovascular vulnerability in this population. Comparison with regional academic populations further contextualizes these findings. A study conducted among university staff in Saudi Arabia reported a high prevalence of cardiometabolic risk factors, including obesity, hypertension, diabetes, and smoking [9]. Together, these findings suggest that academic staff in the Gulf region may constitute a potentially high-risk occupational group requiring targeted prevention strategies.

This study provides important insights into cardiovascular risk among the University of Sharjah employees aged ≥40 years by assessing both clinical and lifestyle factors within a clearly defined occupational population. This approach, combined with an appropriate cross-sectional design, allowed for efficient estimation of cardiovascular risk and its association with lifestyle factors. Stratified random sampling enhanced representativeness across occupational groups, reducing selection bias, while the use of standardized risk tools, including the pooled cohort equations via the Mayo Clinic ASCVD Risk Estimator, ensured methodological rigor and comparability with international guidelines. Focusing on modifiable lifestyle behaviors such as smoking, physical activity, and dietary habits provides clinical and public health relevance, particularly given the limited research on occupational populations like university employees in this region.

Despite these strengths, several methodological limitations should be acknowledged. The cross-sectional design precludes causal inference, and directional interpretations between lifestyle behaviors and cardiovascular risk should be made cautiously. The modest sample size, while sufficient for descriptive and bivariate analyses, precluded multivariable regression and effect size calculation, limiting assessment of potential confounding. Furthermore, reliance on self-reported clinical and behavioral data, including lipid values, physical activity, and dietary intake, introduces the possibility of recall bias, misclassification, and measurement error. Dietary and physical activity variables were assessed using broad categories rather than detailed quantitative measures, potentially limiting sensitivity to detect associations. Finally, conducting the study at a single institution may limit generalizability to other populations. These limitations highlight the need for cautious interpretation and future studies with larger, multicenter, and longitudinal designs using standardized measures.

Overall, the high predicted cardiovascular risk observed in this cohort underscores the need for structured workplace-based screening and preventive interventions. Given the substantial modifiable component of cardiovascular risk [4], targeted lifestyle modification programs that focus on smoking cessation, physical activity promotion, dietary optimization, and metabolic risk management may meaningfully reduce the long-term cardiovascular burden in this population.

## Conclusions

In conclusion, faculty and staff at the University of Sharjah represent a high-risk population with several modifiable risk factors associated with higher predicted ASCVD risk, including smoking, diabetes, and physical inactivity. Given the cross-sectional design and modest sample size, these findings should be interpreted cautiously. Nonetheless, the elevated mean 10-year risk score, coupled with the modifiable nature of key associated factors, highlights the importance of institutional preventive strategies. Targeted workplace interventions, including cardiovascular risk screening, smoking cessation support, and environments that promote physical activity, may be beneficial for addressing factors associated with increased cardiovascular risk within this population.
